# Potential link between caffeine consumption and pediatric depression: A case-control study

**DOI:** 10.1186/1471-2431-11-73

**Published:** 2011-08-25

**Authors:** Cássia R Benko, Antonio C Farias, Lucilene G Farias, Erico F Pereira, Fernando M Louzada, Mara L Cordeiro

**Affiliations:** 1 Department of Neuropsychopharmacology - Pelé Pequeno Príncipe Research Institute, Av. Silva Jardim 1632, Curitiba, 80250-200 PR, Brazil; 2 Faculdades Pequeno Príncipe, Av. Iguaçu, 333, Curitiba, 80230-020, PR, Brazil; 3 Department of Psychology, Children's Hospital Pequeno Príncipe, Av. Desembargador Motta 1070, Curitiba, 80250-060, PR, Brazil; 4 Department of Neuropediatrics, Children's Hospital Pequeno Príncipe, Av. Desembargador Motta 1070, Curitiba, 80250-060, PR, Brazil; 5 Department of Physiology, Federal University of Parana, Curitiba, 81531-990, PR, Brazil; 6 Department of Psychiatry and Biobehavioral Sciences of the David Geffen School of Medicine, Semel Institute for Neuroscience and Human Behavior, University of California Los Angeles, 700 Westwood Plaza, Los Angeles, 90025, CA, USA

## Abstract

**Background:**

Early-onset depressive disorders can have severe consequences both from developmental and functional aspects. The etiology of depressive disorders is complex and multi-factorial, with an intricate interaction among environmental factors and genetic predisposition. While data from studies on adults suggest that caffeine is fairly safe, effects of caffeine in children, who are in period of rapid brain development, are currently unknown. Furthermore, systematic research addressing the relationship between depressive symptoms in children and caffeine consumption is lacking.

The present study examined the effects of caffeine consumption on depressed mood in children with depression and non-depressed participants.

**Methods:**

Children and adolescents (n = 51) already enrolled in an ongoing longitudinal study, aged 9-12 years, were assessed for depressive symptoms with the Children Depressive Inventory (CDI). Psychopathological symptoms were assessed with the Child Behavioral Checklist (CBCL) and eating habits were assessed with the Nutrition-Behavior Inventory (NBI) [1]. The children were compared to control children without psychopathology attending public schools in a Southern Brazilian city.

**Results:**

Participants with CDI scores ≥ 15 (mean = 19; S.D. = 4) also had high NBI scores (mean = 52; S.D. = 19, p < 0.001) suggestive of a relationship between depressive symptoms and environmental factors, in this case nutrition/behavior. Additional linear regression adjusted statistical analysis, considering the factors of consumption of sweets and caffeine individually, showed that caffeine, but not sweets, was associated with depressive symptoms.

**Conclusions:**

These findings indicate that depressed children consume more caffeinated drinks than non-depressed children. Nonetheless while a strong association between depressive symptoms and caffeine consumption among children was found, further research should investigate whether or not this association is due to a cause and effect relationship.

## Background

The etiology of childhood depression is complex and not fully understood. Depressive disorders can result from an interaction between neurobiological, genetic, psychological and social factors. There is a growing body of evidence that environmental variables in combination with genetics may play a fundamental role in the development of this disorder.

There has been a steady increase in caffeine consumption among youngsters over the years with an estimated 75-98% consuming at least one caffeinated beverage daily [2,3]. However, few empirical studies have addressed the possible link between consumption of caffeinated drinks and the development of behavioral and mood disorders in children [3]. Studies have shown that on average children and adolescents consume two 12 oz cans of soda daily [4,5], each containing 20 to 55 mg of caffeine. In addition, there is a new market for youth-directed "energetic drinks" which stimulate both the peripheral and central nervous systems. The usage of these drinks and the psychiatric and long term consequences particularly in children and adolescents needs to be addressed by empirical research.

Prior studies have found that adolescents with caffeine dependence report a greater level of anxious and depressive symptoms [6,7]. A recent study reported that youths diagnosed with major depressive disorder consumed more caffeine and complained of more sleep problems than youths without depression [8]. To further address this issue we examined whether children with depression consumed more caffeine containing beverages than matched healthy controls.

## Methods

### Participants

The present investigation was part of an ongoing longitudinal clinical assessment of behavioral, mood, and learning disorders conducted in our Research Institute [9,10]. This study included 51 children recruited from local public schools, 1^st ^to 5^th ^grade. Through lectures at the public schools, teachers were encouraged to identify students with suspected learning difficulties, mood disorders, attention problems, and hyperactive behavior. The teachers were also informed about the availability of research evaluations via referral to our Research Institute.

Boys often display more externalizing problems, such as aggressive and defiant behaviors, and therefore tend to be clinically referred by teachers more often than girls [11]. Thus, our sample was composed of mostly males (84% male vs. 16% female). Furthermore, all of the subjects were from the same public schools as well as the same socio-economic (lower middle) class. Thus, it is reasonable to assume that confounding lifestyle variables should be minimal. Parents accompanying their children provided written informed consent and the children entered a multi-stage, multi-method assessment process to establish diagnostic groups.

### Procedures and Assessments

Depressive symptoms were measured using the Children Depression Inventory (CDI). The CDI is a widely utilized and well-studied clinical and research tool in the assessment of depressive symptoms in children and adolescents from seven to 17 years of age. The CDI is self-administered and contains 27 items. Responses to each item range from 0-2, 0 = absence of the symptom, 1 = moderate symptom, and 2 = severe symptom [12]. A total CDI score of ≥15 was used as the cut-off score for major depressive disorder [13]. Additional information regarding depressive symptoms as well as the presence of other psychopathologies was obtained through the Child Behavior Checklist (CBCL) [14]. A computer based CBCL was used to convert raw scores to age and gender-standardized T scores. A T score ≥ 70 indicates 98^th ^percentile of the normative sample [14].

Participants received thorough medical evaluations that included neurological, ophthalmological and audiological examinations to rule out perceptual disorders. Full-scale IQ was measured using the Wechsler Intelligence Scale for children, 3^rd ^edition [15]. Subjects with a full-scale IQ ≤ 70 that were < 9 years of age were not included in the study. Inclusion criteria for the depressive group included meeting full DSM-IV-TR criteria [16] and the absence of medication. The controls were children who did not meet criteria for any DSM psychopathology, and who had no other physical conditions, such as epilepsy.

Assessment of dietary behavior was obtained using the Nutrition-Behavior-Inventory (NBI) [1,17]. The NBI is a pencil-and-paper test, with 52 questions used in the assessment of the relationship between aspects of nutrition (in particular eating habits) and psychological symptoms of behavior. Responses are made in a likert format (0-never, 1-occasionally, 2-often, and 3-always). A total NBI score ≥ 30 has been suggested as an indicator of an association between psychological symptoms and eating habits. In addition to analyzing total NBI score, we also analyzed three specific questions regarding consumption of sweets and caffeine containing drinks.

The study protocol was reviewed and approved by our research Ethics Committee on Human Research at the Little Prince Children's Hospital, Curitiba, Brazil.

### Statistical Analyses

The data were analyzed using the software Statistica-7.0 (*StatSoft^®^South America) *using the Student's *t*-test to compare data from children with a CDI≥15 versus a CDI <15. An analysis of covariance (ANCOVA) and multiple linear regression analysis were performed to control for possible confounding variables regarding the association of consumption of caffeinated drinks and depressive symptoms and consumption of sweets. Additionally, the Fisher's exact test was used to determine the differences between the higher and lower consumption groups. The statistical significance level was set at p < 0.05.

## Results

The clinical and demographic characteristics of the groups are presented in Table 1. No significant differences were found in age and sex distribution between the depression and control groups. The Full-scale WISC-III IQ scores did not differ significantly between the depressed group and the controls (p = 0.32). Children with depression presented significantly higher T scores on CBCL scale Internalizing, Externalizing, and Total Problems (p < 0.005).

**Table 1:** Demographic and Clinical Characteristics of the Sample: Means ± Standard Deviation

	***DEPRESSION***	***CONTROLS***	***p***
	***n = 34***	***n = 17***	
Gender (% boys)	88.2%	76.5%	0.30
Age (years)	9.9 (1.0)	9.9 (1.4)	0.86
NBI	52.4 (18.8)	26.1 (12.6)	0.0001*
CDI	19.7(4.0)	9.9 (1.4)	0.0001*
WISC-III FS IQ	95.1 (16.0)	108.1 (24)	0.32
CBCL (T score)			
Internalizing Problems	67.2 (11.6)	58.9 (7.5)	0.005*
Externalizing Problems	69.4 (9.3)	56.1 (8.0)	0.0001*
Total Problems	71.4 (7.1)	58.6 (5.4)	0.0001*

NBI: Nutrition Behavior Inventory; CDI: Children Depression Inventory; WISC-III FS IQ: Wechsler Intelligence Scale for Children, 3rd Ed. Full Scale IQ; CBCL: Children Behavior Checklist; *statistically significant.

**Figure 1:**
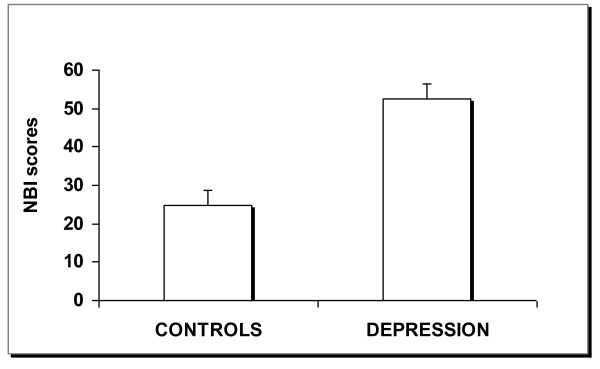
**Children who met diagnostic criteria for Depression also had higher scores on Nutrition Behavior Inventory-NBI (p < 0.001)**.

Children with higher CDI scores (n = 34, 19.7 ± 4, mean ± SD), who met the diagnostic criteria for depression, showed higher NBI scores (52 ± 19, mean ± SD) (p < 0.001) (Figure 1). When ANCOVA analyses were carried out considering sugar consumption, caffeine consumption, and sugar/caffeine consumption as covariates, the difference persisted (p < 0.001). However, after adjusted statistical analyses, in linear regression, considering the factors of sweet and caffeine consumption separately, only caffeine consumption was associated with depressive symptoms (R^2 ^= 0.530; β = 1.640; p = 0.031).

**Figure 2:**
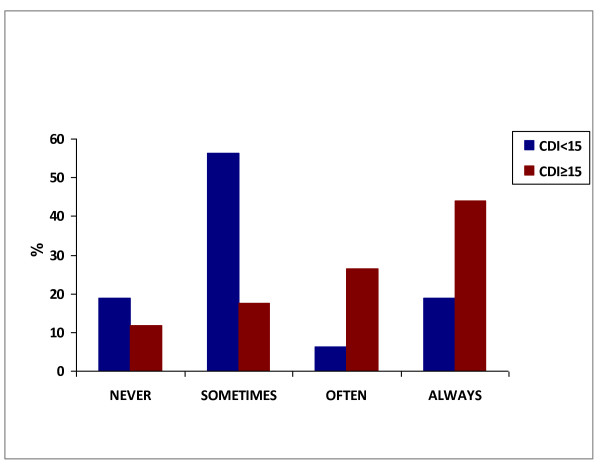
**NBI question #27 "I drink more than 3 cups of coffee, cola or tea per day"; Categories were compared by means Fisher's exact test**. The "never" category was significantly different from each of the other categories (p < 0.001).

The association between caffeine consumption and NBI scores is depicted in Figure 2. All children with caffeine consumption classified as "often" or "always" had CDI scores higher than 15. The comparison between children who reported never drinking caffeine versus those who had caffeine consumption classified as "sometimes", "often", or "always" showed that the percentage of children with a CDI score <15 was significantly higher in the group without caffeine consumption (p < 0.001).

## Discussion

The present study found an association between the consumption of caffeinated drinks and clinical depression in children. However, a cause-effect relationship cannot be determined due to the correlational nature of the data. In fact, very few empirical studies have previously addressed this issue. We cannot determine whether the high consumption of caffeine in our study population was actually "causing" depression or if high consumption was used to relieve some symptoms of depression. It is possible that children meeting diagnostic criteria for major depression use caffeine to "self-medicate" to ease the symptoms associated with depression. Studies have shown that adults often use substances to alleviate psychiatric symptoms [18]. In addition, caffeine usage has been moderately associated with a genetic predisposition for major depression, anxiety and substance use disorders [19]. It is therefore possible that the depressed youth in the current study may ingest caffeine and sweets for temporary relief [7].

The issue of a link between externalizing problems and depression, may be less intuitive. However, studies have shown that depressive symptoms in children are present somewhat differently than in adults. Children frequently show "irritability and aggressiveness", which may be perceived as "externalizing behaviors" by parents and teachers; thus their CBCL-T-scores on the externalizing scale may be high [20,21].

The primary vehicle for caffeine consumption in children is soft drinks, which generally also contain a large amount of sugar [22]. Accordingly, in this study we also found a correlation between depression and the consumption of sweets and colas. Studies have shown that sugar, like caffeine, activates dopaminergic reward circuits in a manner that is similar to drugs of abuse such as cocaine [23-25]. Therefore caffeine in soft drinks may act synergistically on the dopaminergic system, with the high levels of sugar in the drinks.

It is well documented that caffeine consumption in adults increases alertness and leads to high cognitive performance [26,27]. However, studies have also shown that high caffeine consumption can trigger anxiety and depressive-like conditions [28,29]. It has been postulated that caffeine at nontoxic doses acts as a competitive antagonist to the adenosine A1 and A2 receptors [30] and studies have shown that adenosine interacts with dopamine DRD2 receptors [31,32] as well as with glutamatergic neurotransmission [33]. Both the dopaminergic and gluatmatergic systems have been implicated in psychiatric disorders (mood disorders). The adenosine A1 receptor subtype is inhibitory while the A2 subtype is facilitatory. Since caffeine acts at both adenosine receptor subtypes, some authors have argued that caffeine at different concentrations may have opposing effects by acting at different receptors subtypes [34]. Other studies suggest a J-shaped caffeine dosage curve as an independent risk factor for suicide [35]. In fact these researchers have shown that caffeine consumption at low to moderate doses may have protective effects. For example, they found that moderate caffeine consumers had a lower risk of suicide, while heavy coffee drinkers (> 8 cups/day) had a greater risk for suicide [35]. Furthermore, other researchers have found that daily caffeine consumption of up to 140 mg is associated with a reduced risk of depression [36].

Studies in school children have shown that high caffeine consumers (> 50 mg/day) had significantly more negative-effect symptoms than low consumers (< 10 mg/day) [37]. More recent studies have also shown that caffeine-dependent adolescents had a significantly higher score on self-reports of anxiety and depression than a non-dependent group [6]. Interestingly, some studies have also linked the abuse of other drugs with caffeine consumption. Bernestein and colleagues (2002) showed that adolescents dependent on marijuana consumed significantly more caffeine than non-marijuana dependent controls. Earlier studies have shown that alcohol, marijuana, and amphetamine abusers have a greater likelihood of coffee drinking by 12 years of age when compared to non-abusers [38]. It is therefore important for parents to closely monitor their children's consumption habits as a possible indicator of mood, behavior, and/or addictive problems on the rise.

The methods used in the current study present a number of limitations. First, we did not measure the actual average daily intake of caffeine or carbohydrates. We estimated consumption based on self-reported items on the NBI which is based on a likert scale. Second, our results should be considered preliminary due to the small sample size. Third, more male subjects than female subjects participated in the study and thus we cannot generalize our results to the young female population.

## Conclusions

In conclusion, our data suggest that depressed children consume more caffeinated drinks than non-depressed children. However, further studies of caffeine consumption in children, especially those who may be predisposed to major depression, are warranted. Furthermore, since previous studies have also suggested that early caffeine consumption may be an indication of later drug abuse, it is worthwhile for parents to monitor the daily eating and drinking habits of their children. The question as to whether high caffeine consumption may actually cause symptoms of depression or ease the symptoms of depression in children remains unknown. However, the results presented here warrant further investigations to prevent this serious psychiatric disorder from developing.

Understanding the relationship of multiple factors associated with childhood depression may lead to the identification of those at risk and the establishment of preventive protocols and early interventions.

## Authors' contributions

MLC is the principal investigator who designed the study. MLC developed the hypothesis, analyzed and interpreted the results with CRB. MLC wrote the manuscript. CRB and LGF applied the WISC-III and questionnaires (NBI and CDI), ACF conducted all the clinical and medical assessments, EFP and FML performed the statistical analysis. All authors read, commented and concur with the submission.

## Pre-publication history

The pre-publication history for this paper can be accessed here:

http://www.biomedcentral.com/1471-2431/11/73/prepub
